# Suicidal behaviors among refugee women in Jordan: post-traumatic stress disorder, social support and post-displacement stressors

**DOI:** 10.1186/s12889-024-20128-1

**Published:** 2024-09-30

**Authors:** Mohamad Adam Brooks, Anindita Dasgupta, Maysa’ Khadra, Ahmad Bawaneh, Neeraj Kaushal, Nabila El-Bassel

**Affiliations:** 1 Silver School of Social Work, New York University, New York, NY USA; 2 Columbia University School of Social Work, New York, USA; 3 University of Jordan School of Medicine, Amman, Jordan; 4 International Medical Corps, Amman, Jordan; 5 Columbia University Mailman School of Public Health, Department of Sociomedical Sciences, New York, USA

**Keywords:** Refugee, Suicidal behaviors, PTSD, Social support, Post-displacement stressors, Jordan, Women

## Abstract

**Background:**

This paper examines the frequency of suicidal behaviors (suicidal ideation or attempt) among a sample of Syrian refugee women living in non-camp settings in Jordan. We asked several questions surrounding suicide and examined the associations between post-traumatic stress disorder (PTSD), social connectedness, post-displacement stressors and suicidal behaviors.

**Methods:**

Participants (*n* = 507) were recruited using a clinic-based systematic sampling from four health clinics throughout Jordan in 2018. We used a multivariable logistic regression to examine the hypothesis of whether positive screening for PTSD (PCL-5), social isolation (have no friends or family members available to help), and greater number of post-displacement stressors (PMLD Checklist) is associated with suicidal behaviors.

**Results:**

Approximately one-tenth (9.86%) of participants surveyed reported suicidal behaviors (suicidal ideation or attempt) in the past six months. Our hypothesis was partially supported. In the adjusted multivariable analyses, screening positive for PTSD [OR:4.02 (95% CI:1.33, 12.15)] increased odds of suicidal behaviors, while having one friend or family member available to help when in need [OR:0.31 (95% CI:0.13, 0.78)] decreased odds of suicidal behaviors. We did not find any associations between the number of post-displacement stressors and suicidal behaviors in the multivariable model.

**Conclusion:**

Agencies and practitioners addressing suicidal behaviors among Syrian refugee women should provide interventions that aim to reduce PTSD symptoms and social isolation. Potential intervention includes screening for mental health symptoms and suicidal behaviors during routine visits with service providers, as well as providing proper mental health and psychosocial support services according to the mapping of available services.

## Background

Suicide is a serious global public health problem that disproportionately affects vulnerable populations, including refugees [1]. There is a dearth of information in the literature about suicide among refugees, especially in Lower-Middle-Income-Countries [2] and countries in the Middle East and North Africa (MENA) region [3]. Lack of good quality country data, stigma associated with suicide, and likely under-reporting and misclassification of suicide, is associated with difficulties conducting research on this topic [4]. Among studies that examined suicidal behaviors among refugees, rates of suicidal behaviors vary, possibly reflecting different methodological approaches [4, 5].

The Syrian refugee crisis remains one of the largest humanitarian crises. With over 6.8 million Syrians forcibly displaced from their country [6], Jordan hosts one of the largest populations of Syrian refugees at 1.3 million individuals [7]. Syrian refugees in Jordan face socioecological mental health risk factors from a variety of post-displacement stressors that include discrimination, isolation, and barriers to health and mental health services [8–10]. Refugee women, additionally, experience disproportionate amounts of displacement stressors [11], which include risk of living in poverty [12], challenges participating in labor market, workplace discrimination, child caring expectations [13], barriers to accessing sexual and reproductive health [14], as well as sexual, physical, and psychological abuse [15], which contribute to greater risk of adverse mental health outcomes [16]. In Jordan, perspectives on suicide include high levels of stigma, low suicide literacy, and negative attitudes towards seeking psychological help [17]. Furthermore, suicide is considered a socially stigmatized topic that may contradict religious beliefs [18, 19].

A limited number of studies have examined suicidal behaviors among Syrian refugees [5, 20, 21]. Existing studies reflect the high levels of suicidal behaviors in this population. A study of 196 Syrian refugees living in non-camp settings in Cairo, Egypt surveyed the mental health status of participants and found high rates of PTSD (33.5%) and suicidal plans or attempts (13.7%) [21]. Another examined PTSD and suicidality in 195 internally displaced Syrians and 111 Syrian refugees in Netherlands and found higher rates of suicidal behaviors among refugees who were internally displaced [22]. Suicidal behaviors have also been reported in refugee youth (ages 10–17 years old), where a study of 339 Syrian refugee children living in Jordan were assessed for depression, resilience and suicidality [23]. The study found 27.7% of children reported suicidal statements, where depression and bullying were associated with suicidality. Lastly, a review of suicide attempts in emergency room admissions in the MENA region found attempted suicides to be predominantly young women [18]. This emphasizes the importance of examining the role of gender in understanding suicidal behaviors. From our knowledge of the extant literature, no previous studies have examined suicidal behaviors of Syrian refugee women in Jordan.

### Suicidal behaviors – theoretical background

The literature generally distinguishes between suicide and suicidal behaviors [24–26]. Suicide is defined as the act of intentionally ending one’s own life, while suicidal behaviors include nonfatal suicidal thoughts and behaviors [24]. Suicidal behaviors can be further classified specifically into three categories [24]. *Suicide ideation* refers to the thoughts of engaging in behavior intended to end one’s life, while *suicide plan* is the formulation of a specific method through which one intends to die. *Suicide attempt* refers to the engagement in potentially self-injurious behavior in which there is at least some intent to die.

Several leading theories provide insight into suicide and suicidal behaviors. These include the psychache theory, interpersonal theory of suicide (IPTS), integrated motivational-volitional theory (IMV), and the Three-Step Theory (3ST). Although these theories are distinct from one another, they also share similar concepts. The psychache theory was first introduced by Shneidman, who proposed psychological or intrapsychic pain are central to suicidal behaviors [27, 28]. Shneidman argued that suicide is caused by psychache, which he defines as “the hurt, anguish, soreness, aching, psychological pain in the psyche, the mind” [28]. Although the concept of psychological pain is complex, psychache theory emphasizes that psychological pain needs to be perceived as unbearable and to surpass the individual’s threshold for enduring and tolerating pain for it to develop into suicide [28].

Another leading theory is the IPTS, which conceptualizes suicide as two interpersonal constructs: thwarted belongingness and perceived burdensomeness. Thwarted belongingness is a sense of alienation from friends, family, and/or important social circles, whereas perceived burdensomeness is the belief that one’s death is worth more than one’s life [29]. The IPTS indicates that the capability for suicidal behavior emerges in response to repeated exposure to physically painful and/or fear inducing experiences [30].

The IMV is another theory that describes suicidal behavior in three distinct phases: pre-motivational phase, motivational phase, and volitional phase. The pre-motivational phase describes how the biopsychosocial context of an individual, which includes vulnerability factors and stressful life events, provides the backdrop to the development of suicidal ideation [31]. The motivational phase is an individual’s response to a stressful situation, where a feeling of defeat and humiliation without a sense of escape contributes to the emergence of suicidal ideation [31]. The volitional phase, which is similar to the IPTS, includes a range of factors in a person’s life that contribute to the increased capability in suicide. These include, but are not limited to, fearlessness of death, access to means, past suicidal history, tolerance to physical pain [31].

There is currently no specific theory or framework that explains suicidal behaviors among refugees and other displaced populations. One leading theory that has been applied in a handful of studies among refugee populations is the Three-Step Theory. The Three-Step Theory (3ST) roots suicide in a “Idea-to-Action” framework [32]. The theory hypothesizes that suicide results from the combination of pain and helplessness. This pain is typically described as emotional or psychological pain. Second, among those experiencing pain and hopelessness, connectedness is a key protective factor against escalating ideation, in that it prevents the escalation of suicidal ideation. Connectedness typically means connection to other people, but can also mean attachment to a job, role, or a perceived sense of purpose that keeps a person interested in living [32]. We decided to frame our study with the 3ST as several studies have shown this theory to be supported in studies that examined suicidal behaviors among displaced populations, specifically among Congolese refugees in Rwanda and North Korean refugees in South Korea [33, 34]. We provided an overview of several leading theories of suicidal behaviors to highlight differences and similarities in concepts.

### Psychological pain

The 3ST highlights how suicidal ideation and behaviors are rooted in psychological pain. This is reflected in extant literature, where having a mental illness such as PTSD or depression is associated with suicidal behaviors [35, 36]. Among refugee populations, exposure to severe trauma and PTSD is a risk factor for suicidal behavior [37]. Existing models of suicidal behaviors suggests PTSD symptoms to be linked with poor occupational and social functioning, leading to greater life impairment, and subsequently suicidal behaviors [35]. An alternative model suggests that suicidal behaviors when associated with depressive symptoms is linked to severe PTSD symptoms [35]. Depression often includes negative cognitive biases and emotion regulation that leads to self-devaluation, guilt, rumination, diminished interest in activities, and detachment from others – all which contribute to feelings of hopelessness, worthlessness and increased psychological pain [38–40]. This PTSD and suicidal behavior relationship is especially pertinent among refugee populations who have significantly higher rate of PTSD than compared to the general population [41].

Several studies have examined the relationship between mental illness and suicidal behaviors among refugees. Among North Korean refugee youth in South Korea, high levels of PTSD symptoms were associated with suicidal ideation [34]. Among Congolese refugees living in Rwanda, poor mental health was associated with suicidal ideation and/or attempts [33]. Among asylum-seekers in Sweden, refugees (including Syrian) who reported moderate to severe levels of emotional distress were much more likely to exhibit suicidal ideation than those with low levels of distress [42]. These study findings highlight the close association between mental health conditions, psychological pain, and suicidal behaviors.

### Connectedness

The 3ST indicates that isolation and lack of connectedness play an important role in the escalation of suicidal behavior. Few studies have examined this relationship among Syrian refugees and other refugee populations. In a systematic review of quantitative and qualitative studies that examined social connection and suicide among immigrants and asylum-seekers in anglosphere countries, social support and social connection played a role in mitigating suicide risk [43]. Among asylum seekers in the United Kingdom, higher levels of self-harm and suicide were reported among detained asylum seekers, where social isolation was found to be associated with suicide [44]. A study of Bhutanese refugees resettled in the United States also found suicidal ideation to be associated with lower levels of social support [45]. Among Syrian refugees resettled in Sweden, exposure to torture was associated with lower social support while higher social support was associated with less likelihood of PTSD [46]. Previous studies that examined refugees with a history of torture have noted this relationship between torture and suicidal behaviors [47]. The study of Syrian refugees in Sweden also found social support to partially mediate the effect of torture on PTSD [46]. Lastly, among Syrian refugee children in Jordan, the experience of bullying was predictive of suicidality, which suggest the important role of social connectedness even among youth [23].

The role of connectedness and wellbeing also extends to connection with the community, organizations, and other forms of resources. One study of Syrian refugees and Jordanians examined the role of social network in empowerment and wellbeing of women [48]. Larger network size was found to be associated with higher social support, empowerment, and motivation to lead. Additionally, women engaged in volunteer work had more diverse social networks and a higher level of resources and psychological well-being [48]. In another study of Syrian refugees in Jordan, the authors examined the mental health of clinic attending adults and found psychological distress to impact engagement to care [49]. The study highlighted the disconnect between the perception and role of clinic-based mental health programs, and the need for agencies to connect with Syrian refugees to improve psychological well-being and reduce suffering. Other connection to resources, including interventions aim to support parenting among Syrian refugees in Lebanon have also shown to reduce caregiver distress [50], while connections to non-governmental organizations have been shown to help with a range of basic services that aid in the wellbeing of refugees [51, 52].

### Post-displacement stressors

Syrian refugees experience a wide range of post-displacement stressors throughout displacement. In Turkey, a study of Syrian refugees displaced in Istanbul found common post-displacement stressors to include worries about family back home, inability to return home in an emergency, and not having permission to work [53]. Study findings suggested post-displacement stressors, in additional to being female, serve as contextual factors that impact psychological wellbeing [53]. A review of psychosocial concerns reported by Syrian refugees in Jordan found psychosocial distress to be exacerbated by a range of post-displacement stressors that include insecure housing, lack of income, and a variety of employment-based stressors [54]. In Lebanon, an in-depth qualitative study of male and female Syrian refugees living in camps explored the impact of long-term displacement. The study found participants experienced a variety of post-displacement stressors that include harsh living conditions, isolation, discrimination and harassment from the host community, all which can impact family dynamics, gender roles, violence against women, mental health, and lead some participants to express a wish to die [55]. These studies emphasize the frequency and wide range of post-displacement stressors that are associated with negative outcomes of psychological wellbeing.

Although the 3ST does not incorporate post-displacement stressors into its theoretical framework, existing literature on refugee suicidal behavior has emphasized the link between post-displacement stressors and suicidal behaviors. A study of treatment-seeking survivors of torture resettled in New York examined a range of post-displacement stressors and found that being female, having not submitted an asylum application, and a history of rape or sexual assault, was associated with suicidal ideation [56]. A study of Bhutanese refugees resettled in the United States highlight how stressors such as unemployment among men, illiteracy and family conflict among women, were associated with feelings of alienation from family and friends [57]. Furthermore, post-displacement stressors have been examined as a strong predictor of suicidal behaviors among multiple forcibly displaced populations [5].

### Study purpose

This paper examines the frequency of suicidal behaviors among 507 clinic-attending Syrian refugee women living in non-camp settings in Jordan. We first examine the frequency of suicidal behaviors among our sample of Syrian refugee women. We then examine the association between PTSD, connectedness (average number of friends and family members available to help when in need), and post-displacement stressors (post-displacement living difficulties) on suicidal behaviors. We hypothesize that screening positive for PTSD, social isolation (have no friends or family members available to help), and greater number of post-displacement stressors are associated with suicidal behaviors. Gaining insight to suicidal behaviors among Syrian refugees is vital, especially as no end is in sight for the Syrian refugee crisis.

## Methods

### Participants & procedure

Data from Women ASPIRE, a quantitative study that examined health inequities of 507 Syrian refugee women living in non-camp settings in Jordan, was used for this study. Participants were recruited from health clinics from four different cities in Jordan: Amman, Ramtha, Mafraq and Zarqa. Partnered health clinics were selected by identifying geographic location with highest concentration of Syrian refugees in Jordan.

Participants were enrolled between April and November 2018 using a clinic-based systematic sampling. Every third or fifth participant (depending on clinic size) seeking health services was screened for eligibility. Recruitment intervals varied as partnered health clinics had different number of Syrian refugees attending their clinics. Eligibility criteria included participants who identified as Syrian refugees, female, 18 years or older, and who did not live in a refugee camp. Exclusion criteria included study participants who showed signs of cognitive impairment and who were unable to successfully complete the Folstein Mini-Mental State examination [58]. Study participants were informed that compensation packages of daily useable goods (valued approximately seven USD) were provided to those who participated in the survey. Recruitment and surveys were completed by trained research assistants in private rooms at participant health clinics. Women who brought children and who did not want their child to be in the private room during the survey were provided with the option to have another member of the research team take care of their child. Research assistants had a bachelor’s in psychology or related field. Participants were informed of study aims, which were described as obtaining general information of the participant and their household, their physical and mental health, past experiences in Syria and Jordan, as well as their quality of life. Questions surrounding mental health and suicidal behavior were asked during the second half of the survey and participants were reminded that they can take a break, refuse to answer any questions, or stop the interview at any time. Additionally, participants who reported any history of suicidal ideation or attempts were offered referral to appropriate mental health services. Surveys were interviewer-administered in Arabic. Study participants were provided with written informed consent. Study protocols were approved by Columbia University Institutional Review Board and Ethics Committee of University of Jordan prior to the start of the study.

## Measures

### Dependent variable

#### Suicidal ideation

measured by asking participants *Have you had thoughts about ending your life or committing suicide in the past six months* (No/Yes).

#### Suicide attempt

measured by asking participants *Have you tried to kill yourself or attempted suicide in the past six months* (No/Yes).

#### Suicidal behaviors

measured by combining any reported suicidal ideation or attempt in the past six months (No/Yes).

### Independent variables

#### Posttraumatic stress disorder

The PTSD Checklist for DSM-5 (PCL-5): a 20-item Likert self-report scale used to measure presence and severity of PTSD symptoms validated in Arabic [59, 60]. Scores were summed, ranging from 0 to 80. Like previous studies, a cutoff score of 23 and higher was used to screen for PTSD [60]. Cronbach’s alpha estimating internal consistency among our sample is acceptable (α = 0.94).

#### Post migration stressors

Post-Migration Living Difficulties (PMLD): a 14-item checklist used to measure the severity of post-migration or post-displacement problems commonly encountered by immigrants, refugees and/or asylum seekers [61, 62]. Common post-displacement living difficulties include poverty, discrimination, poor access to psychological services, family separation, not being able to find work, poor access for schooling for children, and worry about no treatment for health problems, to name a few. Adapted versions of this checklist have been used [10, 63]. The PMLD scale was dichotomized [64, 65] and participants who rated a PMLD as a Big or Very Big problem were compared to those who rated it as No Problem at all, Moderate Problem, or Not Applicable. The PMLD scale asked common post-displacement problems participants faced in Jordan. A PMLD cumulative score was also created by combing the fourteen dichotomous PMLD items. Scores were added, ranging from 0 to 14. Cronbach’s alpha estimating internal consistency among our sample is acceptable (α = 0.79).

#### Social support from friends or family members

Social support questions examined the type of social support (emotional, informational, and instrumental support) received as well as the social network of participants surveyed. Emotional support includes support based on reassurance of worth, empathy and affection, while instrumental support is based on tangible or material aid received. Informational support is based on advice, guidance and feedback received [66]. Questions were adapted to the Syrian refugee context from several studies that examined social support and social network of women [67–70].

Social support was measured by asking participants the number of friends or family members they currently have. Emotional support questions include the number of friends or family members they currently have: (1) to talk to when feeling upset, angry, or need help, and (2) to talk about relationship problems they are having with spouse. Informational support questions include the number of friends or family members they currently have: (3) to borrow money when in need, (4) to ask to stay at their place for a while, and (5) to ask for tasks such as helping take care of kids. Instrumental support questions include the number of friends and family members they currently have: (6) to ask for advice about personal problems, (7) to ask for legal support, and (8) to ask for help to find a job. This social support measure did not discriminate whether social support received were from friends or family members who lived in Jordan, Syria, or globally.

The *average number of friends or family members* was measured by averaging the number of friends or family members participants reported in their eight different situations of need. The Stata *round* function was used to round the average number of friends or family members to a whole number. Scores were categorized according to participants who reported (0) zero friends or family members, (1) one friend family member, and (2) two or more friends or family members.

### Covariates

Sociodemographic characteristics including age (continuous variable in years), marital status (unmarried/married), number of children < 18 years old in the household (continuous), years of education (continuous), years in Jordan (continuous), and clinic location (Amman/Zarqa/Mafraq/Ramtha) was used in our adjusted model as previous literature linked these sociodemographic characteristics with suicidal behaviors.

The Center for Epidemiological Studies Depression Scale (CES-D): a four-item Likert scale used to measure self-reported symptoms of depression validated in Arabic [71, 72]. Scores were summed, ranging from 0 to 12. A cutoff score of four or higher was used to screen for depression [71]. Cronbach’s alpha estimating internal consistency among our sample is acceptable (α = 0.79). We included the CES-D in our adjusted model, as previous literature highlighted the association between depression and suicidal behaviors among traumatized refugee populations [37].

### Data analysis

Descriptive statistics were used to examine sociodemographic characteristics of participants, frequency distribution and average scores of suicidal behaviors, mental health symptoms severity, post-displacement stressors, and the number of friends or family members who can help in need. Independent sample t-tests were used to calculate significant mean differences for continuous variables between dependent and independent variables, while Chi-square tests or Fisher exact tests was used to calculate significant differences between categorical variables of dependent and independent variables.

A multivariable logistic regression model was used to test our hypothesis that examined the association between the average number of friends or family members who can help in need, PTSD cutoff score, post-displacement stressors (cumulative score), and suicidal behaviors. Adjusted covariates for the multivariable model include age, marital status, number of children < 18 in household, years of education, years in Jordan, clinic location, and depression cutoff score. We did not utilize multilevel modeling to account for nested data as the interclass correlation coefficient (ICC) across clinics was close to zero [73]. We also assessed multicollinearity in both our unadjusted and adjusted multivariable models using the variance inflation factor (VIF) measure. We did not find any evidence of multicollinearity as our VIF score for each individual variable was less than two [74]. All analyses were completed using STATA (version 15.1) [75].

## Results

### Characteristics of the sample

Characteristics of participants are presented in Table 1. A total of 507 Syrian refugee women were surveyed from four health clinics throughout Jordan: Amman (30.18%), Ramtha (25.25%), Mafraq (24.85%), and Zarqa (19.72%). The mean age was 34.15 years old (SD = 10.99). Most women were married (90.14%) and the mean number of children under 18 years-old was 3.39 (SD = 2.08). The average number of years of education was 7.09 (SD = 3.67). Most participants originated from As-Suwayda, Daraa or Qunitra (35.9%) and the mean number of years lived in Jordan was 5.18 (SD = 1.38).

**Table 1:** Characteristics of Syrian Refugee women living in non-camp settings in Jordan (*n* = 507)

	*n*	x̄ (SD) or %
Age (SD)		34.15 (10.99)
Marital Status		
Unmarried Married	50 457	9.86 90.14
Children under 18 in household		3.39 (2.08)
Years of education	503	7.09 (3.67)
Years in Jordan		5.18 (1.38)
Clinic Location		
Amman Zarqa Mafraq Ramtha	153 100 126 128	30.18 19.72 24.85 25.25
Governorate of Origin in Syria		
Aleppo or Idlib	96	18.93
Al-Raqqah, Deir et-Zor, or Hasaka Damascus or Rif Dimashq As-Suwayda, Daraa or Qunitra	54 60 182	10.65 11.83 35.90
Hama or Homs	115	22.68

Note. n = sample size; x̄ = sample mean; SD = standard deviation; % = percentage

### Suicidal behaviors

Approximately one-tenth of women surveyed (9.07%) reported suicidal ideation within the past six months (Table 2). Forty-nine (9.66%) reported history of a suicide attempt, while fourteen (2.76%) reported a suicide attempt within the past six months. Approximately one-tenth (9.86%) of women reported a suicidal behavior (suicidal ideation and/or attempt) in the past six months. We found several significant bivariate associations among suicidal behaviors (Table 3). A significantly higher proportion of participants who reported suicidal ideation (34.78%) or suicide attempt (100.00%) in the past six months reported a history of suicide attempts. Additionally, a significant higher proportion of participants who reported a suicide attempt in the past six months reported suicidal ideation in the past six months (71.43%).

**Table 2:** Suicidal behaviors and mental health symptoms

Variables	*n*(%) or x̄(SD)
History of suicide attempt	
No Yes	458 (90.34) 49 (9.66)
Suicidal ideation past six months	
No Yes	461 (90.93) 46 (9.07)
Suicidal attempt past six months	
No Yes	493 (97.24) 14 (2.76)
Suicidal behaviors past six months (suicidal ideation and/or attempt)	
No Yes	457 (90.14) 50 (9.86)
Received any mental health services in the past six months	
No Yes	437 (86.19) 70 (13.81)
PCL-5 Symptom severity score	32.74 (19.36)
PCL-5 Average item score	1.64 (0.97)
PCL-5 Cutoff Score (*n* = 506)	
Screen negative for PTSD (Cutoff < 23) Screen positive for PTSD (Cutoff ≥ 23)	171 (33.79) 335 (66.21)
CES-D Symptom severity score	5.32 (3.65)
CES-D Average item score	1.33 (0.91)
CES-D Cutoff Score	
Screen negative for Depression (Cutoff < 4) Screen positive for Depression (Cutoff ≥ 4)	188 (37.08) 319 (62.92)

Note. n = sample size; x̄ = sample mean; SD = standard deviation; % = percentage

PCL-5 = PTSD Checklist for DSM-5

CES-D = Center for Epidemiological Studies Depression scale

**Table 3:** Bivariate analysis between suicidal behaviors and mental health symptoms

		Suicidal Ideation past six months (*n* = 507)			Suicide Attempts past six months (*n* = 507)		
	Total (*n* = 507)	Participants who did not report Suicidal Ideation (*n* = 461)	Participants who reported Suicidal Ideation (*n* = 46)	Chi-Square or T-test	Participants who did not report Suicide Attempts (*n* = 493)	Participants who reported Suicide Attempts (*n* = 14)	Chi-Square or T-test
	n(%) or x̄(SD)	n(%) or x̄(SD)	n(%) or x̄(SD)		n(%) or x̄(SD)	n(%) or x̄(SD)	
History of suicide attempt							
No Yes	458 (90.34) 49 (9.66)	428 (92.84) 33 (7.16)	30 (65.22) 16 (34.78)	***p*** ** < 0.001**	458 (92.90) 35 (7.10)	0 (0.00) 14 (100.00)	***p*** ** < 0.001**
Suicidal ideation past six months							
No Yes	461 (90.93) 46 (9.07)	- -	- -	-	457 (92.70) 36 (7.30)	4 (28.57) 10 (71.43)	***p*** ** < 0.001**
PCL-5 Symptoms Severity Score (*n* = 506)	32.74 (19.36)	31.19 (18.85)	48.26 (17.67)	***p*** ** < 0.001** ^b^	32.36 (19.28)	46.29 (17.93)	***p*** ** < 0.008** ^b^
PCL-5 Cutoff Score (*n* = 506)							
Screen negative for PTSD (Cutoff < 23) Screen positive for PTSD (Cutoff ≥ 23)	171 (33.79) 335 (66.21)	166 (97.08) 5 (2.92)	294 (87.76) 41 (12.24)	***p*** ** < 0.001** ^a^	169 (98.83) 2 (1.17)	323 (96.42) 12 (3.58)	***p*** ** = 0.155** ^a^
CES-D Symptom Severity Score	5.32 (3.65)	5.09 (3.57)	7.59 (3.71)	***p*** ** < 0.001** ^b^	5.23 (3.61)	8.36 (3.73)	***p*** ** = 0.002** ^b^
CES-D Cutoff Score							
Screen negative for depression (Cutoff < 4) Screen positive for depression (Cutoff ≥ 4)	188 (37.08) 319 (62.92)	179 (38.83) 282 (61.17)	9 (19.57) 37 (80.43)	***p*** ** = 0.010**	186 (37.73) 307 (62.27)	2 (14.29) 12 (85.71)	***p*** ** = 0.094** ^a^

Note. n = sample size; x̄ = sample mean; SD = standard deviation; % = percentage; PCL-5 = PTSD Checklist for DSM-5; CES-D = Center for Epidemiological Studies Depression scale

Chi-square test used unless otherwise noted; significant *p*-values (*p* < 0.05) are highlighted in bold

^a^ Fisher’s exact test used; ^b^ T-test used

### Mental health symptoms

Among participants surveyed, approximately two-thirds (66.21%) screened positive for PTSD. PTSD symptom score ranged from 0 to 78 (not shown), with a mean of 32.74 (SD = 19.36). Less than two-thirds (62.92%) of women surveyed screened positive for depression. Depression symptom score ranged from 0 to 12 (not show), with a mean of 5.32 (SD = 3.65). Several significant associations were found in our bivariate analysis (Table 3). Participants who reported suicidal ideation in the past six months had, on average, a higher PTSD symptoms severity score (*p* < 0.001) and a higher depression symptoms severity score (*p* < 0.001) than compared to those who did not report suicidal ideation in the past six months. Participants who reported suicidal attempts in the past six months had, on average, a higher PTSD symptoms severity score (*p* = 0.008) and depression symptom severity score (*p* = 0.002) than compared to those who did not report suicidal attempts. Furthermore, a higher proportion of participants who screen positive for PTSD (*p* < 0.001) and those who screen positive for depression (*p* = 0.010) reported suicidal ideation in the past six months. No significant associations between participants who reported suicide attempts in the past six months and PTSD or depression cutoff scores were found. We also found most participants surveyed (86.19%) did not receive any form of mental health services in the past six months.

### Post-displacement stressors

PMLD scores ranged from 0 to 13 (not shown), with a mean score of 7.43 (SD = 2.98) (Table 4). The most common Big/Very Big post-displacement stressor experienced was Poverty (79.09%). We found several significant bivariate associations (Table 4). A higher proportion of participants who reported Loneliness and Boredom (*p* = 0.001), Isolation (*p* = 0.005), or Family Separation (*p* = 0.044) as a Big/Very Big Problem reported suicidal ideation in the past six months. We also found a higher proportion of participants who reported Loneliness and Boredom (*p* < 0.001) and Isolation (*p* = 0.033) as a Big/Very Big Problem reported suicide attempts in the past six months. Similarly, a higher proportion of participants who reported Loneliness and Boredom (*p* < 0.001), Isolation (*p* = 0.003), and Family Separation (*p* = 0.025) as a Big/Very Big Problem reported suicidal behaviors in the past six months. Lastly, participants who reported suicidal ideation (*p* = 0.014) or suicidal behaviors (*p* = 0.006) in the past six month had on average, a higher PMLD total score than compared to those who did not report suicidal ideation or suicidal behaviors. No significant relationships between suicide attempts in the past six months and PMLD total score was found.

**Table 4:** Bivariate analysis between post-displacement stressors (PMLD) and suicidal behaviors

		Suicidal Ideation past six months (*n* = 507)			Suicide Attempts past six months (*n* = 507)			Suicidal Behaviors past six months (*n* = 507)		
	Total (*n* = 507)	Participants who did not report Suicidal Ideation (*n* = 461)	Participants who reported Suicidal Ideation (*n* = 46)	Chi-Square or T-test	Participants who did not report Suicide Attempts (*n* = 493)	Participants who reported Suicide Attempts (*n* = 14)	Chi-Square or T-test	Participants who did not report Suicidal Behaviors (*n* = 457)	Participants who reported Suicidal Behaviors (*n* = 50)	Chi-Square or T-test
	n(%) or x̄(SD)	n(%) or x̄(SD)	n(%) or x̄(SD)		n(%) or x̄(SD)	n(%) or x̄(SD)		n(%) or x̄(SD)	n(%) or x̄(SD)	
PMLD Total (SD) *n* = 503	7.43 (2.98)	7.33 (3.01)	8.47 (2.49)	***p*** ** = 0.014** ^b^	7.39 (2.98)	8.71 (2.84)	***p*** ** = 0.103** ^b^	7.31 (3.00)	8.53 (2.55)	***p*** ** = 0.006** ^b^
Poverty (%)										
Big/Very Big Problem No/Moderate Problem	401 (79.09) 106 (20.91)	361 (78.31) 100 (21.69)	40 (86.96) 6 (13.04)	***p*** ** = 0.189**	390 (79.11) 103 (20.89)	11 (78.57) 3 (21.43)	***p*** ** = 1.000**	358 (78.34) 99 (21.66)	43 (86.00) 7 (14.00)	***p*** ** = 0.206**
Fears of forced return to Syria (%) *n* = 506										
Big/Very Big Problem No/Moderate Problem	399 (78.85) 107 (21.15)	364 (79.13) 96 (20.87)	35 (76.09) 11 (23.91)	***p*** ** = 0.630**	389 (79.07) 103 (20.93)	10 (71.43) 4 (28.57)	***p*** ** = 0.508**	361 (79.17) 95 (20.83)	38 (76.00) 12 (24.00)	***p*** ** = 0.603**
Worry no treatment for health problem (%) *n* = 506										
Big/Very Big Problem No/Moderate Problem	377 (74.51) 129 (25.49)	340 (73.91) 120 (26.09)	37 (80.43) 9 (19.57)	***p*** ** = 0.333**	365 (74.19) 127 (25.81)	12 (85.71) 2 (14.29)	***p*** ** = 0.534**	337 (73.90) 119 (26.10)	40 (80.00) 10 (20.00)	***p*** ** = 0.348**
Worry of Family in Syria (%)										
Big/Very Big Problem No/Moderate Problem	372 (73.37) 135 (26.63)	337 (73.10) 124 (26.90)	35 (76.09) 11 (23.91)	***p*** ** = 0.662**	361 (73.23) 132 (26.77)	11 (78.57) 3 (21.43)	***p*** ** = 0.769**	333 (72.87) 124 (27.13)	39 (78.00) 11 (22.00)	***p*** ** = 0.436**
Unable to Return Home in Emergency (%) *n* = 506										
Big/Very Big Problem No/Moderate Problem	365 (72.13) 141 (27.87)	330 (71.74) 130 (28.26)	35 (76.09) 11 (23.91)	***p*** ** = 0.531**	357 (72.56) 135 (27.44)	8 (57.14) 6 (42.86)	***p*** ** = 0.229**	326 (71.49) 130 (28.51)	39 (78.00) 11 (22.00)	***p*** ** = 0.330**
Not being able to find work (%) *n* = 504										
Big/Very Big Problem No/Moderate Problem	361 (71.63) 143 (28.37)	324 (70.59) 135 (29.41)	37 (82.22) 8 (17.78)	***p*** ** = 0.119**	352 (71.84) 138 (28.16)	9 (64.29) 5 (35.71)	***p*** ** = 0.553**	322 (70.77) 133 (29.23)	39 (79.59) 10 (20.41)	***p*** ** = 0.193**
Poor access to schooling for children (%)										
Big/Very Big Problem No/Moderate Problem	318 (62.72) 189 (37.28)	287 (62.26) 174 (37.74)	31 (67.39) 15 (32.61)	***p*** ** = 0.492**	308 (62.47) 185 (37.53)	10 (71.43) 4 (28.57)	***p*** ** = 0.585**	284 (62.14) 173 (37.86)	34 (68.00) 16 (32.00)	***p*** ** = 0.416**
Loneliness and Boredom (%) *n* = 506										
Big/Very Big Problem No/Moderate Problem	289 (57.11) 217 (42.89)	252 (54.78) 208 (45.22)	37 (80.43) 9 (19.57)	***p*** ** = 0.001**	275 (55.89) 217 (44.11)	14 (100.00) 0 (0.00)	***p*** ** < 0.001**	248 (54.39) 208 (45.61)	41 (82.00) 9 (18.00)	***p*** ** < 0.001**
Isolation (%) *n* = 503										
Big/Very Big Problem No/Moderate Problem	251 (49.90) 252 (50.10)	219 (47.92) 238 (52.08)	32 (69.57) 14 (30.43)	***p*** ** = 0.005**	240 (49.08) 249 (50.92)	11 (78.57) 3 (21.43)	***p*** ** = 0.033**	216 (47.68) 237 (52.32)	35 (70.00) 15 (30.00)	***p*** ** = 0.003**
Family Separation (%)										
Big/Very Big Problem No/Moderate Problem	248 (48.92) 259 (51.08)	219 (47.51) 242 (52.49)	29 (63.04) 17 (36.96)	***p*** ** = 0.044**	239 (48.48) 254 (51.52)	9 (64.29) 5 (35.71)	***p*** ** = 0.286**	216 (47.26) 241 (52.74)	32 (64.00) 18 (36.00)	***p*** ** = 0.025**
Poor access to psychological services (%)										
Big/Very Big Problem No/Moderate Problem	194 (38.26) 313 (61.74)	171 (37.09) 290 (62.91)	23 (50.00) 23 (50.00)	***p*** ** = 0.086**	188 (38.13) 305 (61.87)	6 (42.86) 8 (57.14)	***p*** ** = 0.783**	170 (37.20) 287 (62.80)	24 (48.00) 26 (52.00)	***p*** ** = 0.136**
Discrimination (%) *n* = 506										
Big/Very Big Problem No/Moderate Problem	82 (16.17) 425 (83.83)	75 (16.27) 386 (83.73)	7 (15.22) 39 (84.78)	***p*** ** = 0.853**	77 (15.62) 416 (84.38)	5 (35.71) 9 (64.29)	***p*** ** = 0.059**	73 (15.97) 384 (84.03)	9 (18.00) 41 (82.00)	***p*** ** = 0.688**
Immigration Application Challenges (%) *n* = 505										
Big/Very Big Problem No/Moderate Problem	80 (15.84) 425 (84.16)	71 (15.47) 388 (84.53)	9 (19.57) 37 (80.43)	***p*** ** = 0.524**	75 (15.27) 416 (84.73)	5 (35.71) 9 (64.29)	***p*** ** = 0.055**	69 (15.16) 386 (84.84)	11 (22.00) 39 (78.00)	***p*** ** = 0.209**
Communication (%) *n* = 506										
Big/Very Big Problem No/Moderate Problem	32 (6.32) 474 (93.68)	28 (6.09) 432 (93.91)	4 (8.70) 42 (91.30)	***p*** ** = 0.519** ^a^	31 (6.30) 461 (93.70)	1 (7.14) 13 (92.86)	***p*** ** = 0.604** ^a^	28 (6.14) 428 (93.86)	4 (8.00) 46 (92.00)	***p*** ** = 0.544** ^a^

Note. n = sample size; x̄ = sample mean; SD = standard deviation; % = percentage; PMLD = Post-Migration Living Difficulties

PMLDs are listed according to greatest frequency of being a Big/Very Big Problem

Chi-square test used unless otherwise noted; significant *p*-values (*p* < 0.05) are highlighted in bold

^a^ Fisher’s exact test used; ^b^ T-test used

### Social support and isolation

Table 5 reflects the average number of friends or family members participants reported when they needed help, with a mean score of 1.42 (SD = 1.04). Over one-tenth (12.18%) of participant reported having no friends or family members to ask for when they needed help, while over half (52.73%) reported having one friend or family members, and more than one-third (35.08%) reported having two or more friends or family members. The most common issue participants reported were having no friends or family members to help with was to ask for legal support (75.64%). We found several significant associations in our bivariate analysis (Table 5). A higher proportion of participants who reported having zero friends to ask for help with a task such as helping care of children, reported suicidal ideation in the past six months (*p* = 0.019), as well as suicidal behaviors in the past six months (*p* = 0.013). Differences between the combined average number of friends and family members and suicidal behaviors were also found (*p* = 0.043).

**Table 5:** Bivariate analysis between suicidal behaviors and number of current friends and family members (FFM)

		Suicidal Ideation past six months (*n* = 507)			Suicide Attempts past six months (*n* = 507)			Suicidal Behaviors past six months (*n* = 507)		
	Total (*n* = 507)	Participants who did not report Suicidal Ideation (*n* = 461)	Participants who reported Suicidal Ideation (*n* = 46)	Chi-Square test	Participants who did not report Suicide Attempts (*n* = 493)	Participants who reported Suicide Attempts (*n* = 14)	Chi-Square test	Participants who did not report Suicidal Behaviors (*n* = 457)	Participants who reported Suicidal Behaviors (*n* = 50)	Chi-Square test
	n(%) or x̄(SD)	n(%) or x̄(SD)	n(%) or x̄(SD)		n(%) or x̄(SD)	n(%) or x̄(SD)		n(%) or x̄(SD)	n(%) or x̄(SD)	
**Number of FFM you can ask for legal support** *n* = 505										
0 friends/family 1 friend/family 2 or more friends/family	382 (75.64) 69 (13.66) 54 (10.69)	346 (75.38) 63 (13.73) 50 (10.89)	36 (78.26) 6 (13.04) 4 (8.70)	***p*** ** = 0.965** ^a^	372 (75.76) 66 (13.44) 53 (10.79)	10 (71.43) 3 (21.43) 1 (7.14)	***p*** ** = 0.641** ^a^	344 (75.60) 61 (13.41) 50 (10.99)	38 (76.00) 8 (16.00) 4 (8.00)	***p*** ** = 0.766** ^a^
**Number of FFM you can ask to help you find a job** *n* = 504										
0 friends/family 1 friend/family 2 or more friends/family	327 (64.88) 67 (13.29) 110 (21.83)	300 (65.50) 63 (13.76) 95 (20.74)	27 (58.70) 4 (8.70) 15 (32.61)	***p*** ** = 0.178** ^a^	318 (64.90) 65 (13.27) 107 (21.84)	9 (64.29) 2 (14.29) 3 (21.43)	***p*** ** = 1.000** ^a^	297 (65.42) 63 (13.88) 94 (20.70)	30 (60.00) 4 (8.00) 16 (32.00)	***p*** ** = 0.151** ^a^
**Number of FFM you can ask to stay at their place for a while** *n* = 504										
0 friends/family 1 friend/family 2 or more friends/family	230 (45.63) 154 (30.56) 120 (23.81)	208 (45.41) 138 (30.13) 112 (24.45)	22 (47.83) 16 (34.78) 8 (17.39)	***p*** ** = 0.550**	224 (45.71) 150 (30.61) 116 (23.67)	6 (42.86) 4 (28.57) 4 (28.57)	***p*** ** = 0.884** ^a^	206 (45.37) 138 (30.40) 110 (24.23)	24 (48.00) 16 (32.00) 10 (20.00)	***p*** ** = 0.801**
**Number of FFM you can talk about relationship problems with spouse** *n* = 448										
0 friends/family 1 friend/family 2 or more friends/family	182 (40.63) 128 (28.57) 138 (30.80)	165 (40.54) 119 (29.24) 123 (30.22)	17 (41.46) 9 (21.95) 15 (36.59)	***p*** ** = 0.552**	176 (40.46) 125 (28.74) 134 (30.80)	6 (46.15) 3 (23.08) 4 (30.77)	***p*** ** = 0.938** ^a^	163 (40.45) 119 (29.53) 121 (30.02)	19 (42.22) 9 (20.00) 17 (37.78)	***p*** ** = 0.349**
**Number of FFM you can ask for help with a task such as helping take care of children** *n* = 505										
0 friends/family 1 friend/family 2 or more friends/family	140 (27.72) 147 (29.11) 218 (43.17)	120 (26.14) 140 (30.50) 199 (43.46)	20 (43.48) 7 (15.22) 19 (41.30)	***p*** ** = 0.019**	132 (26.88) 145 (29.53) 214 (43.58)	8 (57.14) 2 (14.29) 4 (28.57)	***p*** ** = 0.065** ^a^	118 (25.93) 139 (30.55) 198 (43.52)	22 (44.00) 8 (16.00) 20 (40.00)	***p*** ** = 0.013**
**Number of FFM you can ask for advice about personal problems** *n* = 503										
0 friends/family 1 friend/family 2 or more friends/family	104 (20.68) 183 (36.38) 216 (42.94)	94 (20.52) 167 (36.46) 197 (43.01)	10 (22.22) 16 (35.56) 19 (42.22)	***p*** ** = 0.964**	100 (20.45) 177 (36.20) 212 (43.35)	4 (28.57) 6 (42.86) 4 (28.57)	***p*** ** = 0.502** ^a^	92 (20.26) 166 (36.56) 196 (43.17)	12 (24.49) 17 (34.69) 20 (40.82)	***p*** ** = 0.786**
**Number of FFM you can ask to borrow money when you need it** *n* = 503										
0 friends/family 1 friend/family 2 or more friends/family	99 (19.68) 148 (29.42) 256 (50.89)	92 (20.13) 134 (29.32) 231 (50.55)	7 (15.22) 14 (30.43) 25 (54.35)	***p*** ** = 0.723**	95 (19.43) 143 (29.24) 251 (51.33)	4 (28.57) 5 (35.71) 5 (35.71)	***p*** ** = 0.452** ^a^	90 (19.87) 132 (29.14) 231 (50.99)	9 (18.00) 16 (32.00) 25 (50.00)	***p*** ** = 0.899**
**Number of FFM you can talk to when you feel upset**,** angry**,** or need help** (SD) *n* = 502										
0 friends/family 1 friend/family 2 or more friends/family	64 (12.75) 142 (28.29) 296 (58.96)	57 (12.47) 129 (28.23) 271 (59.30)	7 (15.56) 13 (28.89) 25 (55.56)	***p*** ** = 0.815**	60 (12.30) 137 (28.07) 291 (59.63)	4 (28.57) 5 (35.71) 5 (35.71)	***p*** ** = 0.079** ^a^	55 (12.14) 128 (28.26) 270 (59.60)	9 (18.37) 14 (28.57) 26 (53.06)	***p*** ** = 0.435**
**Combined average number of FFM** (*continuous*) *n* = 476	1.42 (1.04)	1.43 (1.06)	1.30 (0.82)	***p*** ** = 0.406**	1.43 (1.05)	1.14 (0.86)	***p*** ** = 0.314**	1.44 (1.06)	1.27 (0.87)	***p*** ** = 0.297**
**Combined average number of FFM** *n* = 476										
0 friends/family 1 friend/family 2 or more friends/family	58 (12.18) 251 (52.73) 167 (35.08)	50 (11.57) 234 (54.17) 148 (34.26)	8 (18.18) 17 (38.64) 19 (43.18)	***p*** ** = 0.114**	55 (11.90) 244 (52.81) 163 (35.28)	3 (21.43) 7 (50.00) 4 (28.57)	***p*** ** = 0.545** ^a^	48 (11.21) 233 (54.44) 147 (34.35)	10 (20.83) 18 (37.50) 20 (41.67)	***p*** ** = 0.043**

Note. n = sample size; x̄ = sample mean; SD = standard deviation; % = percentage

Number of friends or family members are listed in descending order according to greatest frequency of having zero friends or family members

Chi-square test used unless otherwise noted; significant *p*-values (*p* < 0.05) are highlighted in bold

^a^ Fisher’s exact test used

### Hypothesis testing – PTSD, social support, and post-displacement stressors

We found significant associations in our multivariable logistic regression (Table 6). Screening positive for PTSD was associated with increased odds of suicidal behaviors in the past six months in the unadjusted model [OR:4.39 (95% CI:1.56, 12.35)] and the adjusted model [OR:4.02, (95% CI:1.33, 12.15)]. Furthermore, having on average one friend or family member to ask for help when needed was associated with a 67% decreased odd of suicidal behaviors in the unadjusted model [OR:0.33 (95% CI:0.14, 0.79)] and a 69% decreased odds of suicidal behaviors in the adjusted model [OR:0.31 (95% CI:0.13, 0.78)] than compared to having on average zero friend or family members to ask for help. We did not find any significant associations between having two or more friends or family members to ask for help with suicidal behaviors. Significant associations between PMLDs and suicidal behaviors were not found in the unadjusted or adjusted multivariable logistic regression. Lastly, age was the only adjusted covariate that was significant in our adjusted multivariable logistic regression. We did not find any significant association between marital status, number of children under 18 in household, years of education, years in Jordan, CES-D, and clinic location on suicidal behaviors in our adjusted multivariable analysis.

**Table 6:** Multivariable association between suicidal behaviors, social support, PTSD, and post-displacement stressors

	Unadjusted Analysis OR (95%CI)	*p*-value	Adjusted Analysis OR (95%CI)	*p*-value
Average number of friend or family member can ask for help when needed				
0 friends or family members	*ref.*		*ref.*	
1 friend or family member 2 + friend or family members	**0.33 (0.14**,** 0.79)** 0.54 (0.23, 1.28)	***p*** **= 0.013** ***p*** **= 0.162**	**0.31 (0.13**,** 0.78)** 0.55 (0.22, 1.38)	***p*** **= 0.013** ***p*** **= 0.203**
PCL-5 Cutoff Score				
Screen negative for PTSD (Cutoff < 23)	*ref.*		*ref.*	
Screen positive for PTSD (Cutoff ≥ 23)	**4.39 (1.56**,** 12.35)**	***p*** ** = 0.005**	**4.02 (1.33**,** 12.15)**	***p*** ** = 0.014**
Post-displacement stressors (PMLDs)	1.04 (0.92, 1.19)	***p*** ** = 0.520**	1.05 (0.91, 1.21)	***p*** ** = 0.500**

Note. OR = odds ratio; *ref*. = reference category; CI = confidence interval

Adjusted covariates were age, marital status, children under 18 in household, years of education, years in Jordan, CES-D, and clinic location

PCL-5 = PTSD Checklist for DSM-5; PMLD = Post-Migration Living Difficulties; CES-D = Center for Epidemiological Studies Depression scale

Significant *p*-values (*p* < 0.05) are highlighted in bold

## Discussion

This study examines the frequency of suicidal behaviors among Syrian refugee women in Jordan. Overall, the rates of suicidal ideation (9.07%) and suicide attempts (2.76%) in this study sample was on the lower range of suicidal ideation (0.17-70.6%) and suicide attempts (0.14-15.1%) found in a systematic review of suicidal behaviors among forcibly displaced populations globally [20]. As highlighted in the systematic review, comparing suicidal behaviors between studies is difficult, as heterogeneity between study populations, varying analytic approaches and sampling frames, and different study designs make it difficult to compare [20]. The review also examined suicidal behaviors among several subgroups of displaced populations, specifically refugees granted asylum, living in temporary camps, asylum seekers, and internally displaced persons. They found refugees granted asylum had a lower risk of suicide than compared to the host population, while those who were asylum seekers had a higher risk of suicide. The relationship between immigration status and suicidal behaviors is highlighted in a study of 1,085 refugees from several different countries (including Syria) living in Australia, where those with insecure visas status (asylum-seeker, temporary visa, bridging visa) were more likely to report thoughts of being better off dead (39.5%) and have suicidal intent (8.1%) than compared to those with secure visa status: thoughts of being better off dead (18.7%) and suicidal intent (1.5%) [76]. The systematic review also found refugees who lived in camps had a higher risk of suicidal ideation than compared to the host population [20]. This is shown in a study of 300 Palestinian refugee women living in refugee camps in Jordan that found rates of suicidal thoughts to be 13.6% while suicidal attempts were 7.3% [77]. These findings provide some explanation to why rates of suicidal behaviors found in our study may be on the lower range, as our study sample included refugees living in non-camp settings who have a greater likelihood to integrate with the host community and access to resources, than compared to refugees with insecure immigration status, or those who live temporary camps with limited resources. It also highlights the importance of local context, and the consideration of other potential factors that may contribute to suicidal behaviors. Study findings, regardless, emphasize the high rates of suicidal behaviors among displaced populations and provide valuable insight to understanding suicidal behaviors among Syrian refugees.

To contextualize our study findings further, mental health, suicide, and suicidal behaviors are topics that are socially stigmatized in Jordan [17, 18]. A study of 707 Arab youth in Jordan recruited from an online self-report survey found participants to have relatively high levels of suicide stigma, low suicide literacy, and negative attitudes towards seeking psychological help [17]. A review of suicide attempts in emergency rooms in the MENA region found attempted suicides were predominantly women who experienced familial or interpersonal stressors [18]. These findings are echoed in a study of 62 individuals in Jordan who were admitted to emergency room settings as a result of self-harm and suicide attempts. The study found that most participants who attempted suicide were women (71%), with the largest precipitating factor being family violence (54.8%), followed by marital disharmony (12.9%) and financial problems (12.9%) [78]. The review also mentioned the mediating role of culture, where strong familial ties of the Arab nuclear family can serve as a protective factor and support for patients with mental illness or suicidal ideation, or rather exacerbate suicidal behaviors if privacy of the nuclear family result in fear of judgement as a result of stigma towards mental illness and suicide [18]. Religion is seen as another mediating factor, where religiosity can serve as a protective factor and a form of coping during life crises that can empower the individual. However, it can also exacerbate suicidal behaviors if an individual feels abandoned by God or if suicide is viewed as unacceptable [18].

Our paper hypothesized that screening positive for PTSD, social isolation (have no friends or family members available to help), and greater number of post-displacement stressors to be associated with suicidal behaviors. We found our hypothesis to be partially supported. We found PTSD and social isolation were associated with greater odds of suicidal behaviors; however, the number of post-displacement stressors was not associated with suicidal behaviors. We also found having on average one friend or family member to ask for help when needed significantly reduced the odds of suicidal behaviors compared to those who had zero friends or family members to ask for help when needed. We did not find significant associations between having on average two or more family members to ask for help when needed and suicidal behaviors.

Several reasons may explain why PTSD was associated with suicidal behaviors. One explanation is the important role psychological pain plays in suicide and suicidal behaviors – as emphasized in the 3ST theory and psychache theory. Almost two-thirds of our participants screened positive for PTSD (66.21%) and depression (62.92%), which is high, even when compared to a systematic review of rates of PTSD (16–84%) and depression (11–49%) among Syrian refugees displaced in neighboring countries. Additionally, our sample focused exclusively on women, which as suggested in a study of Syrian refugees residing in Turkey, is a risk factor for PTSD [79]. Their study found combined PTSD rates of men and women in their sample to be 35%; however, the probability of PTSD increased to 71% if they were female gender, had a previous psychiatric disorder, a family history of psychiatric disorders, and experienced 2 or more traumas [79]. Furthermore, forced displacement is associated with multiple stressors and traumas for many women [11], which include sexual, physical, and psychological abuse [15]. This, in addition to the protracted crisis may prolong psychological pain and therefore exacerbate feelings of hopelessness and suicidal behaviors.

Additionally in our bivariate analysis, we found suicidal ideation to be significantly associated with both PTSD and depression symptom severity and cutoff scores (screening positive for PTSD or depression). However, for suicide attempts, PTSD and depression were not associated with cutoff scores, but only with symptom severity. This provides evidence that symptom severity and psychological pain may also contribute to the escalation from suicidal ideation to suicide attempts, as supported in the psychache theory. Our study also found history of suicidal attempts, suicidal ideation and suicidal attempts in the past six months to be significantly associated with one another, supporting the close association between suicidal ideation and attempts as suggested in the “Idea-to-Action” framework of the 3ST theory [32].

Several reasons may explain why social isolation was associated with suicidal behaviors. One reason is that social support – or having friends or family members available to ask for help when needed – may act as a buffer to stressful events [80]. This is especially relevant, as strong familial ties is an important factor in the Arab nuclear family, and where family violence and marital disharmony has been seen as a leading precipitating factor in suicidal attempts in emergency room settings [18, 78]. Syrian refugee women who are socially isolated may not be able to find support to reduce levels of psychological pain. This is also reflected in the 3ST theory of ideation-to-action framework, where connectedness acts as a protective factor against escalation of suicidal ideation [32]. Connectedness has been found to be associated with suicidal behaviors in previous studies of displaced populations, including asylum seekers in the United Kingdom as well as Bhutanese refugees resettled in the United States [44, 45]. One possible reason why having two or more friends or family members available to ask for help was not associated with suicidal behaviors may be because our study did not assess the quality of the social support received.

One possible reason why the number of post-displacement stressors was not associated with suicidal behaviors may be related to the severity and type of post-displacement stressor and its impact on daily life stress, which may have a greater association with suicidal behaviors. A study of North Korean refugee women living in South Korea highlights the role of stress, where those who attempted suicide were more likely to have higher level of stress than compared to those who reported suicidal ideation only [81]. Furthermore, the type of post-displacement stressor may play a contextual role in suicidal behavior, where not having submitted an asylum application, unemployment, family conflict was associated with suicidal ideation or feelings of alienation [56, 57]. Furthermore, PMLD total scores were only significantly associated with suicidal ideation, and not suicide attempts, suggesting that post-displacement stressors may contribute to the development of suicidal ideation, but that other factors may contribute to the escalation to suicide attempts.

### Limitations

Our analysis presents several limitations. Studies on suicide are challenging due to the small number of respondents who report suicidal behaviors. Our study is no different. The small sample size limited the ability to complete multivariable analysis for suicide ideation and attempts separately. The cross-sectional design of this study limits the ability to draw causal inferences. Future studies should include longitudinal studies and to included validated instruments to assess suicidal behaviors among Syrian refugees. We also examined the number of friends and family participants had in their social network but did not assess the quality of the support received. We attempted to address the inability to measure the quality of their social network by focusing on social isolation (no friends or family in network). Future studies should examine both the number and quality of friends and family in their social network on suicidal behaviors. Furthermore, utilizing a multivariable analysis may not have provided a comprehensive picture of the relationship between suicidal behaviors, PTSD, social support, and postmigration stressors. It is possible that in future studies, other analyses such as Structural Equation Modelling, may provide a nuanced explanation of this relationship. Additionally, our study did not ask participants’ religion, therefore we were unable to assess the role of religion in relationship to suicidal behaviors. Future studies should examine how religion is associated with suicidal behaviors, mental health, and coping among Syrian refugee women. Mental health and suicidal behaviors remain topics that are culturally stigmatized in Jordan and may have contributed to lower rates of suicidal behaviors found in our study. We attempted to reduce this stigma and fear of discrimination by working closely with mental health experts to ensure that measures be as culturally appropriate as possible. Our research assistants were trained to be non-judgmental and culturally sensitive to mitigate social desirability bias in our study. Lastly, our study sample focuses entirely on Syrian refugee women who are in early and middle adulthood, which limits the generalizability of our findings to any other age group or gender.

### Implications

Suicide continues to be a serious global public health issue that disproportionally affects vulnerable populations, especially among immigrants and refugees [82]. Few studies have examined suicidal behaviors among Syrian refugees despite it being the largest humanitarian crisis of our time. Our paper highlights the need for future research to examine the contextual risk and protective factors associated with suicidal behaviors among this population that increase levels of psychological pain and decrease social connectedness.

This study highlights the close relationship between PTSD, social connectedness, and suicidal behaviors. Clinicians and organizations working with Syrian refugees must screen for a range of mental health conditions (including PTSD) and suicidal behaviors during routine visits and refer to proper mental health and psychosocial support (MHPSS) services that are available. Clinicians and organizations can also enhance social support systems for those who are isolated by providing group counseling or other interventions to enhance their support system. A recent systematic review of individuals at risk for suicide highlighted the efficacy of psychosocial group treatment and peer connection to increase social connectedness and to decrease suicide symptoms [83]. Policy makers should also advocate to reduce the stigma surrounding suicide, as recent laws passed in Jordan have indicated fines and prison sentences for those who attempt suicide [84]. Future studies should also examine suicidal behaviors and risk factors among a variety of different demographics, which include Syrian refugee youth, older adults, and across genders, so that interventions can be tailored and contextualized accordingly.

## Data Availability

The datasets generated and/or analyzed during the current study are not available publicly due to the sensitivity of the context and as data is pertaining to refugees. Datasets remain available from the corresponding author upon reasonable request.

## Abbreviations

3ST: Three-Step Theory of suicide
ASPIRE: Advancing solutions in policy, implementation, research, and engagement for refugees
CES-D: Center for epidemiological studies depression scale
CI: Confidence Interval
DSM-5: Diagnostic and Statistical Manual of Mental Disorders Fifth Edition
ICC: Interclass Correlation Coefficient
IMV: Integrated Motivational-Volitional theory of suicide
IPTS: Interpersonal Theory of Suicide
MENA: Middle East and North Africa
MHPSS: Mental Health and Psychosocial Support
OR: Odds ratio
PCL-5: The PTSD checklist for DSM-5
PMLD: Postmigration living difficulty checklist
PTSD: Post-traumatic stress disorder
SD: Standard deviation
USD: The United States Dollar
VIF: Variance Inflation Factor
